# Revisiting the High-Pressure Behaviors of Zirconium: Nonhydrostaticity Promoting the Phase Transitions and Absence of the Isostructural Phase Transition in *β*-Zirconium

**DOI:** 10.3390/ma16145157

**Published:** 2023-07-21

**Authors:** Lei Liu, Qiumin Jing, Hua Y. Geng, Yinghua Li, Yi Zhang, Jun Li, Shourui Li, Xiaohui Chen, Junjie Gao, Qiang Wu

**Affiliations:** National Key Laboratory of Shock Wave and Detonation Physics, Institute of Fluid Physics, CAEP, Mianyang 621900, China; s102genghy@caep.cn (H.Y.G.); li_yinghua@126.com (Y.L.); zhangyishp@126.com (Y.Z.); lijun102@caep.cn (J.L.); junjie0510@126.com (J.G.); wuqianglsd@163.com (Q.W.)

**Keywords:** Zr, phase transition, high pressure, nonhydrostaticity

## Abstract

Zirconium (Zr) is an important industrial metal that is widely used in nuclear engineering, chemical engineering, and space and aeronautic engineering because of its unique properties. The high-pressure behaviors of Zr have been widely investigated in the past several decades. However, the controversies still remain in terms of the phase transition (PT) pressures and the isostructural PT in β-Zr: why the PT pressure in Zr is so scattered, and whether the β to β’ PT exists. In the present study, to address these two issues, the Zr sample with ultra-high purity (>99.99%) was quasi-hydrostatically compressed up to ~70 GPa. We discovered that both the purity and the stress state of the sample (the grade of hydrostaticity/nonhydrosaticity) affect the PT pressure of Zr, while the stress state is the dominant factor, the nonhydrostaticity significantly promotes the PT of Zr. We also propose two reasons why the β-β’ isostructural PT was absent in the subsequent and present experiments, which call for further investigation of Zr under quasi-compression up to 200 GPa or even higher pressures.

## 1. Introduction

Zirconium (Zr), possessing a low neutron absorption cross-section, hardly absorbs neutrons, thus leading to its being widely used in the nuclear industry. In addition, Zr is protected by a thin oxide layer, making it highly resistance to corrosion, which is a great advantage in chemical engineering. Furthermore, Zr is used to manufacture high-temperature parts in space and aeronautic industries because it is highly resistant to heat. In the past decades, the high-pressure behaviors of Zr have been widely investigated, especially its phase transitions (PT) at high pressures [1,2,3,4,5,6,7,8,9,10,11,12,13,14,15]. Zr stabilizes in a hexagonal closed-packed (hcp) lattice at ambient conditions, known as the α phase. The α phase transforms to the ω phase with a hexagonal structure (an AlB_2_-type structure with Z = 3). By further compression, Zr adopts the body-centered cubic (bcc) structure, referred to as the β phase. However, there are two longstanding controversies in the PT of Zr at high pressure:

(1) The quite scattered PT pressures of Zr. The α-ω PT pressure is as low as 1.2 GPa [12], while it reaches as high as 17 GPa [14], varying more than 10-fold. The onset of the ω-β PT also varies from 28 GPa [2] to 35 GPa [14]. Zhao et al. considered the PT pressure was sensitive to the chemical composition of Zr [16]. However, the relationship between the sample purity and the PT pressure remains vague. In addition, it seems the stress state of the sample (i.e., the grade of hydrostaticity/nonhydrostaticity) also affects the PT pressure [14]. Consequently, what effects play the dominant role in these PTs is still unclear to date, although it is critical to understand the PT mechanism of Zr.

(2) The isostructural PT in β-Zr. Akahama et al. [3] and Stavrou et al. [10] claimed that they discovered an isostructural β-β’ PT in β-Zr: the β’ phase had the same structure as the β phase while it possessed a remarkable volume collapse of 4% during the phase transition, originating from the anharmonic effects. This first-order isostructural PT is of great interest to the condensed matter physics community because this phenomenon in pure elements is extremely rare [17,18,19]. However, this isostructural PT has not been determined in the subsequent experiments reported by Pigott et al. [13] and Anzellini et al. [14], whether from the experimental or theoretical side. Therefore, whether this β-β’ PT exists remains controversial to date.

In this study, we revisit these two controversies by quasi-compressing the ultra-high purity (>99.99%) of Zr up to ~70 GPa. We discover that both the sample purity and nonhydrostaticity affect the PT pressure of Zr, and nonhydrostaticity is the dominant factor. The β-β’ isostructural PT is absent in the present study. We propose two possible reasons to explain the absence of the isostructural PT in β-Zr.

## 2. Experimental Details

The synchrotron-based XRD experiments were performed at the 15U1 beamline of Shanghai Synchrotron Radiation Facility (SSRF). A pair of diamond anvils with culets size of 300 μm was mounted on symmetric cells to generate high pressures. The Re gasket with an initial thickness of 250 μm was indented to around 30 μm. A hole was drilled in the center of the pre-indented region and served as a sample chamber. Several pieces of Zr samples with purity >99.99% were scraped off the ingots. One piece of Zr was subsequently loaded in the sample chamber with a small piece of a Cu pressure gauge [20]. Liquid argon was loaded in the sample chamber serving as pressure transmitting media (PTM) using the cryogenic method. Monochromatic X-ray with a wavelength of 0.6199 Å (20 keV) was first collimated to several micrometers and then illuminated on the sample. The diffraction signal was collected by a MAR165 CCD (2048 × 2048 pixels with a pixel size of 79 × 79 μm^2^) detector. The CeO_2_ reference from the National Institute of Standards and Technology (NIST, Gaithersburg, MD, USA) was used to calibrate the distance from the sample to detector and the tilt of the detector. The 2D diffraction images were integrated by Dioptas [21] to acquire the 2*θ*-intensity curves, which were further analyzed by the Le Bail method implemented in the GSAS and EXPGUI software packages [22].

## 3. Results and Discussions

### 3.1. Phase Transition

**Figure 1:**
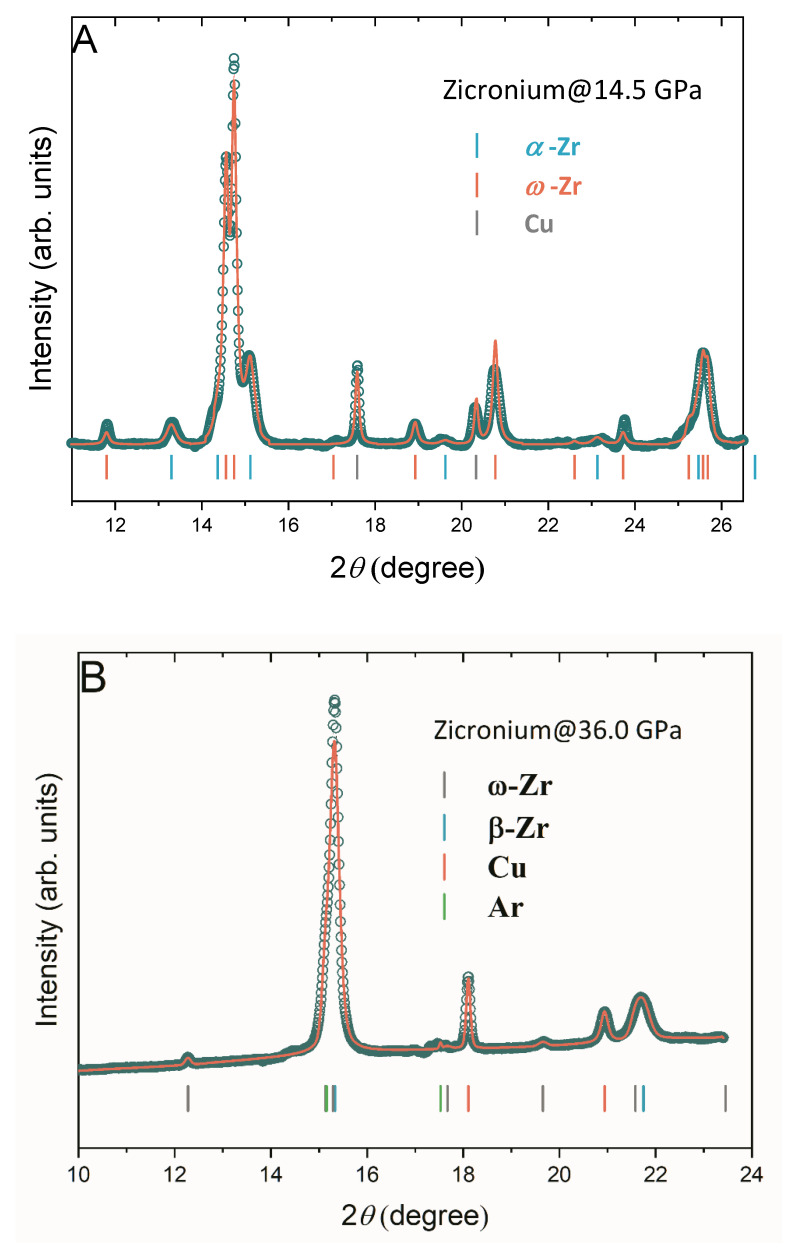
Selected XRD patterns of Zr at high pressure. (**A**) The XRD pattern collected at 14.5 GPa demonstrates the coexistence of the α- and ω-Zr. (**B**) The XRD pattern collected at 36.0 GPa shows the commencement of the ω-β phase transition of Zr. The asymmetric feature of the strongest peak originates from the existence of the Ar (PTM) reflection on the left shoulder.

In the present study, the diffraction patterns of the quasi-hydrostatically compressed Zr were collected up to 67.8 GPa. The reflections from ω-Zr appear at 14.5 GPa (see Figure 1A, orange tick marks), indicating the commencement of the α-ω PT in Zr. This onset pressure of α-ω PT is close to another quasi-hydrostatic result of 17 GPa recently reported by Anzellini et al. [14], while it is more than 10-fold higher than the onset pressure (1.2 GPa) measured in the shear experiment [12]. Considering both Pandey et al. [12] and the present study used the ultra-high purity of the Zr sample, this demonstrates that the sample purity is not the dominant factor that affects the α-ω PT pressure of Zr. The other factors, e.g., the stress state of the sample, have to be taken into account.

**Figure 2:**
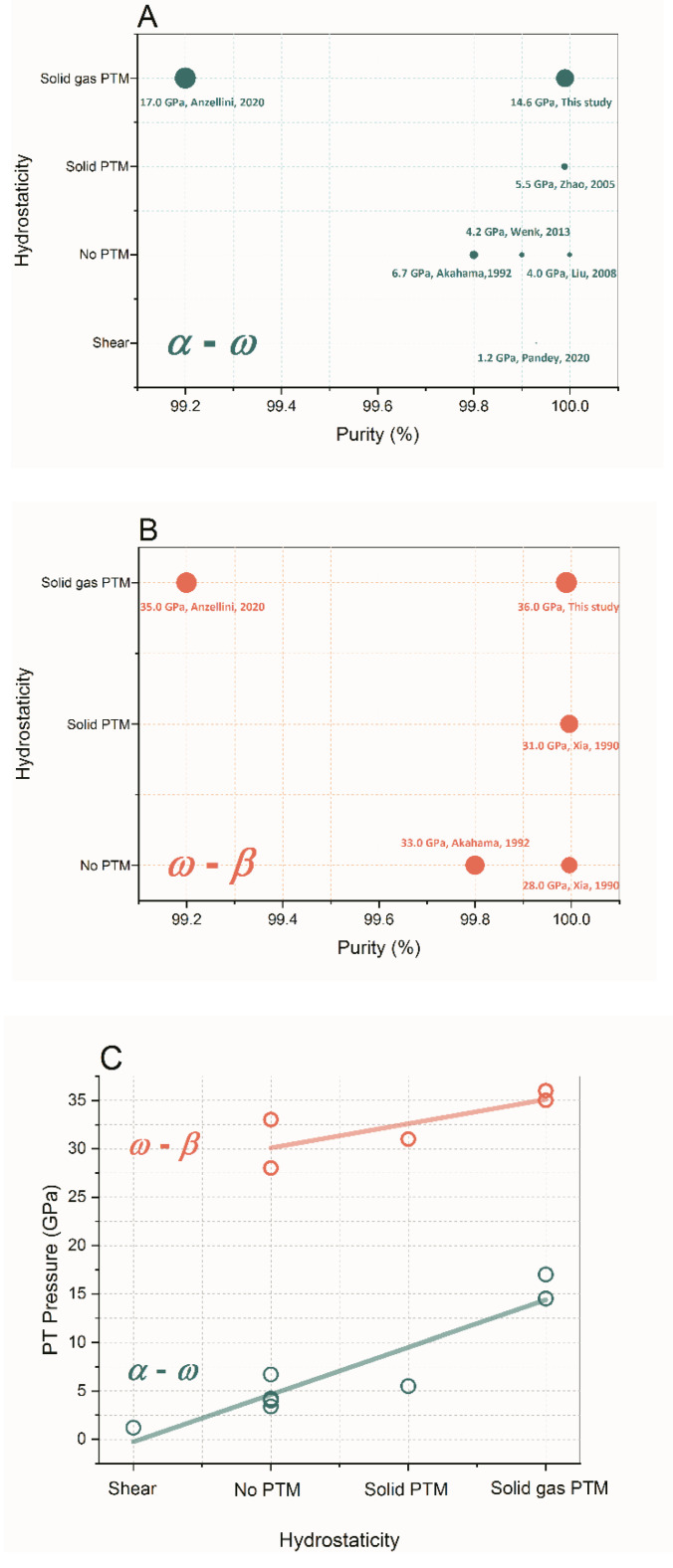
Summary of the onset pressures of the α-ω and ω-β phase transitions of Zr with various purities in different stress environments. The size of the symbols in (**A**,**B**) denotes the values of pressure. (**A**) The α-ω phase transition pressure. (**B**) The ω-β phase transition pressure. (**C**) The PT pressures increase as the grade of hydrostaticity increases. The solid lines are a visual guide.

In Figure 2A and Table 1, we summarize the α-ω PT pressures of Zr with various purities under different stress environments. The symbol size represents the value of the PT pressure. It is unambiguous that both sample purity and hydrostaticity affect the α-ω PT pressure of Zr, but the hydrostaticity is the dominant factor; nonhydrostaticity (especially the shear stress) dramatically promotes the α-ω PT from 17 GPa [14] to as low as 1.2 GPa [12] (see Figure 2C). The non-zero non-diagonal elements of the stress matrix, i.e., the shear stresses, lower the stability of the hcp lattice of Zr. Considering the experiments conducted without PTM (Akahama et al. [3], Liu et al. [8], and Wenk et al. [9], i.e., at the same grade of hydrostaticity), the α-ω PT pressure of Zr decreases (from 6.7 GPa to 4.0 GPa) as sample purity increases. This conclusion is in line with the results reported previously by Zhao et al. [16]: the onset pressure of the α-ω PT in impure Zr with 1.03 at% Hf and 4.5 at% O was higher than that of ultra-pure Zr. They found that a large quantity of O ions in the impure Zr affected the transformation mechanism and stability of the hcp lattice of Zr. A similar relationship between the α-ω PT pressure and the purity of bulk Zr sample under dynamic compression was also discovered by Rigg et al. [23].

Figure 1B shows the X-ray diffraction pattern of Zr collected at 36 GPa. Several reflections from β-Zr (cyan tick marks) are present, indicating the beginning of the ω-β PT. The asymmetric feature of the strongest peak is attributed to the presence of the Ar (PTM) reflection on the left shoulder. In Figure 2B, the onset pressure of the ω- to β-Zr PT is also summarized. It was also found that the PT pressure increased by more than 25% (from 28 GPa to 36 GPa) as the hydrostaticity of the experiment increased. The effect of hydrostaticity on the ω-β transition is not as salient as that in the α-ω PT (See Figure 2C). Considering the experiments conducted without PTM (Akahama et al. [3] and Xia et al. [2], i.e., at the same grade of nonhydrostaticity), the ω-β PT pressure of Zr decreases (from 33 GPa to 28 GPa) as the sample purity increases.

Summing up the results presented above, the following conclusions are drawn: (1) both the sample purity and nonhydrostaticity affect the PT pressure of Zr; (2) the PT pressure decreases as the sample purity increases; and (3) nonhydrostaticity significantly promotes the PT transition of Zr and represents the dominant factor that affects the PT pressure of Zr.

### 3.2. Absence of Isostructural Phase Transition of β-Zr

**Figure 3:**
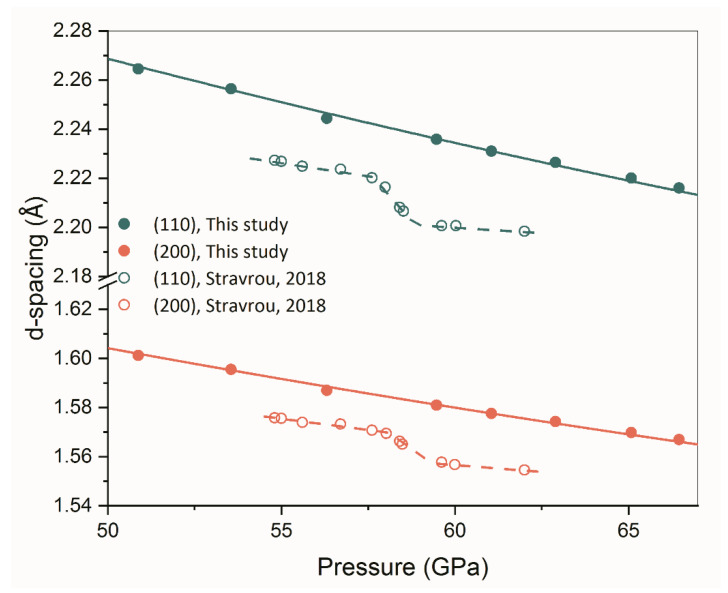
The *d*-spacing of β-Zr as a function of pressure. No kinks were observed in the present results. The solid lines are the polynomial fitting of the present data points and the dashed lines are visual guides. The data points from Stravrou et al. [10] showing abnormal decreases in the pressure range of 55–60 GPa are also shown for comparison.

Akahama et al. [3] and Stavrou et al. [10] claimed that they discovered an isostructural PT in β-Zr under non-hydrostatic compression, i.e., from β to β’ phase accompanying a volume collapse of ~4%. To examine whether this β-β’ PT exists, we plot the *d*-spacings of (110) and (200) reflections of β-Zr as a function of pressure in Figure 3.

**Figure 4:**
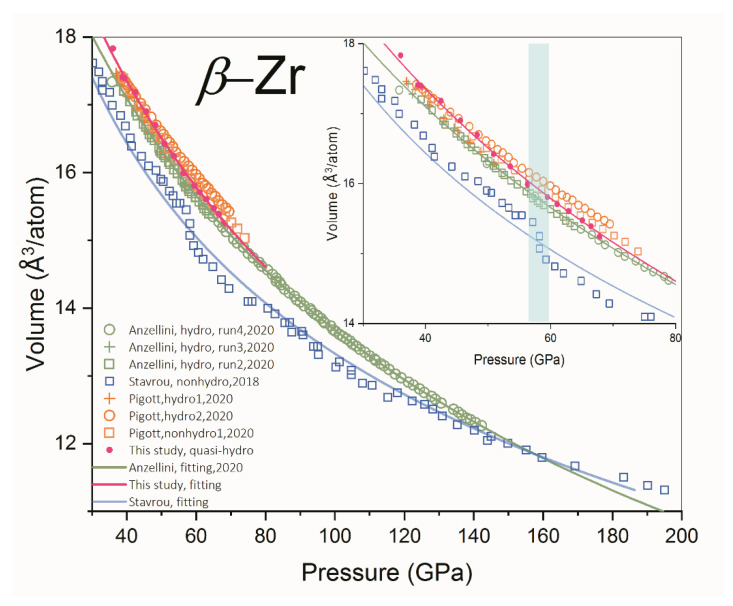
Equation state of β-Zr. The quasi-hydrostatic data from Anzellini et al. [14], the nonhydrostatic data from Stavrou et al. [10], and the quasi-hydrostatic and nonhydrostatic data from Pigott et al. [13] are also included for comparison. The blue solid line is the fitting line of P-V data obtained by Stavrou et al. [10], excluding the data points in the pressure range of 55–80 GPa. The inset shows the data points in the pressure range of 30–80 GPa. The blue zone indicates the pressure range where the volume collapse happened as reported by Stavrou et al. [10].

The present results show that the *d*-spacings of the two reflections mentioned above decrease monotonically and smoothly as pressure increases, without any of the kinks that were discovered by Stavrou et al. [10] in the pressure range of ~58–60 GPa. The P-V data of the present study are plotted in Figure 4 together with the results from Stavrou et al. [10], Pigott et al. [13], and Anzellini et al. [14]. Generally, our results agree well with those of Pigott et al. [13] and Anzellini et al. [14], while Stavrou et al. [10] obtained a pronouncedly lower volume than the other sets of data. This is opposite to our observation of the nonhydrostatic effect on unit cell volume measurement. In axial diffraction geometry, the diffraction vector is close to the radial direction of DAC. The stress along the radial direction is smaller than that along the axial direction because of the uniaxial compression character of DAC. Consequently, the measured *d*-spacing and the unit cell volume of the crystal under nonhydrostatic compression is larger than that measured under hydrostatic compression [24,25,26,27]. All sets of data, except that from Stavrou et al. [10], reveal that there is no volume collapse in β-Zr in the pressure range of 58–60 GPa. The parameters of the equation of state (EOS) of β-Zr are summarized in Table 2. The discrepancies among the bulk modulus may arise from the different pressure scales used and the different grades of hydrostaticity in the experiments.

Why is the previously reported isostructural PT in β-Zr absent in the present study? We consider there are two possible reasons: (1) the isostructural PT does not exist at high pressure. The abnormal behavior of the P-V data in the pressure range of 55–80 GPa obtained by Stavrou et al. [10], recognized as the β-β’ isostructural PT, arose from the pressure difference between the sample and the pressure marker. The blue solid line in Figure 4 is the fitting result of P-V data obtained by Stavrou et al. [10], excluding the data points in the pressure range of 55–80 GPa. This line well represents the high-pressure behavior of β-Zr up to 200 GPa, except for the pressure range of 55–80 GPa. Taking this line as a reference, the measured pressure in the pressure range of 55–80 GPa is only ~5 GPa lower. The reason for this is the relative position between Zr and the pressure marker changes with the deformation and plastic flow of the gasket and sample under high pressure. Without PTM, the pressure gradient (∂P/∂r) in the sample chamber is determined by the strength of Zr: $\frac{\partial P}{\partial r}=\frac{Y}{h}$ [28,29], where *Y* and *h* are the yield strength and thickness of Zr, respectively. At high pressure, the pressure gradient can reach GPa/μm [30,31]. Consequently, the relative position between Zr and the pressure marker changes of several μm can lead to a pressure change of ~5 GPa. (2) The isostructural PT does exist, but the pressure range of the present study is not sufficiently high enough. As we discussed above, nonhydrostaticity has the ability to dramatically lower the PT pressure of Zr. Thus, a much higher pressure, perhaps larger than 142 GPa (the highest pressure achieved in quasi-hydrostatic compression by Anzellini et al. [14]), is needed to trigger the possible β-β’ PT if it does exist. This hypothesis calls for further investigation on Zr under quasi-hydrostatic compression to ~200 GPa or even higher.

## 4. Conclusions

In the present study, Zr with ultra-high purity was quasi-hydrostatically compressed to ~70.0 GPa. The α-ω and ω-β PTs were determined at ~14.5 GPa and 36.0 GPa, respectively. Both the purity of the sample and the hydrostaticity affect the PT of Zr: the nonhydrostaticity promotes the PT and the PT pressure decreases as sample purity increases. No isostructural PT was determined in β-Zr in the present study because of two possible reasons. First, the isostructural PT does not exist, and the abnormal volume collapse discovered by Stavrou et al. [10] arises from the underestimation of the pressure of the sample. Second, the isostructural PT does exist, while the quasi-hydrostaticity hinders the PT. Thus, a hydrostatic experiment up to 200 GPa is necessary, as discussed in V [32].

## Figures and Tables

**Table 1:** The onset pressure of phase transition in Zr at high pressures.

Reference	Purity	PTM	*α*-*ω* (GPa)	*ω*-*β* (GPa)	*β*-*β*’ (GPa)
[2]	99.996%	No		28	
		ME *		31	
[3]	99.8%	No	6.7	33	53
[5]	99.989%	NaCl	5.5		
[8]	99.9995%	No	4.0		
[9]	99.9%	No	3.4–4.2		
[10]	99.5%	No			58
[14]	99.2%	He/Ne	17	35	
This study	99.99%	Ar	14.5	36	

* The mixture of methanol and ethanol (1:4 in volume).

**Table 2:** Vinet EOS parameters of β-Zr. The bold values were fixed during the fitting procedure.

Reference	*V*_0_ (Å^3^/atom)	*K*_0_ (GPa)	*K’*_0_
[10]	**22.6**	80	3.35
[10]	22.5	43	**6**
[10] *	**22.6**	26	6.08
[14]	**22.6**	93	3.20
This study	**22.6**	112	2.28

* Using all the data points except those in the pressure range of 55–80 GPa.

## Data Availability

The data presented in this study are available on request from the corresponding author.
